# Addition of intratympanic steroid or hyperbaric oxygen treatment to systemic steroid treatment in sudden idiopathic sensorineural hearing loss treatment, and long-term results of salvage treatment

**DOI:** 10.3906/sag-1908-122

**Published:** 2020-02-13

**Authors:** Kemal KESEROĞLU, Gökhan TOPTAŞ, Ahmet ULUAT, Ömer BAYIR, Emel Çadallı TATAR, Güleser SAYLAM, Mehmet Hakan KORKMAZ, Ali ÖZDEK

**Affiliations:** 1 Department of Otolaryngology, University of Medical Sciences, Dışkapı Yıldırım Beyazıt Training and Research Hospital, Ankara Turkey; 2 Department of Otolaryngology, Balıkesir State Hospital, Balıkesir Turkey; 3 Department of Otolaryngology, Faculty of Medicine, Yıldırım Beyazit University, Ankara Turkey; 4 Private Clinic, Ankara Turkey

**Keywords:** Idiopathic sudden sensorineural hearing loss, systemic steroid treatment, hyperbaric oxygen therapy, intratympanic steroid treatment, salvage treatment

## Abstract

**Background/aim:**

This study aims to determine the therapeutic superiority of the addition of intratympanic steroid or hyperbaric oxygen therapy to systemic steroid treatment in idiopathic sudden sensorineural hearing loss as initial treatment, and evaluate the long-term results of salvage treatment.

**Materials and methods:**

This study was a retrospective clinical trial with a total of 96 patients with idiopathic sudden sensorineural hearing loss. Patients were divided into 3 groups. Group 1 (n: 32) received systemic steroid treatment. Group 2 (n: 32) received the Group 1 protocol plus intratympanic steroid treatment. Group 3 (n: 32) received the Group 1 protocol plus hyperbaric oxygen treatment. Pretreatment and postinitial audiologic evaluations were performed, and the hearing outcome was analyzed with Furuhashi criteria. All patients, except those who experienced total recovery after initial treatment, were directed to salvage treatment. Audiologic assessment was performed again after salvage treatment and a mean follow-up period of 36.5 months.

**Results:**

Each group was homogenous according to demographics, audiologic data, and prognostic factors. There was no statistically significant difference in recovery and success rate within the 3 groups after initial treatment. (P: 0.66, P: 0.248, respectively). Successful results were obtained after salvage treatment in only 3 patients (5%). These patients received follow-up treatment at a mean of 36.5 months, but there was no spontaneous recovery after the end of salvage treatment.

**Conclusion:**

The addition of intratympanic steroids or hyperbaric oxygen to systemic steroids caused no significant hearing improvement as the initial treatment of idiopathic sudden sensorineural hearing loss. The efficacy of salvage treatment was limited, and there was no spontaneous hearing improvement after the long-term follow-up.

## 1. Introduction

Idiopathic sudden sensorineural hearing loss (ISSHL) is an infrequent otologic emergency which is defined as loss of more than 30 dB in at least 3 contagious frequencies within 3 days. Up-to-date ISSHL etiology, pathogenesis, and treatment have not been clarified exactly. Systemic steroids (SS) are the most frequently used treatment modality that has been investigated in several clinical trials [1,2]. There are still controversies in administration route, dosage, duration of therapy, and selection of corticosteroid type. After the first attempt of intratympanic steroid (ITS) usage in inner ear disease in 1991, 3 different protocols of ITS usage in ISSHL have been developed: initial monotherapy, initial adjunctive to SS, and salvage therapy. However, the best dosage, type, and timetable are still unclear today [3,4]. Hyperbaric oxygen therapy (HBOT), which is frequently used as an alternative modality to steroids in the treatment of ISSHL, diminishes cochlear hypoxia, edema, and damage. Efficacy in ISSHL treatment is still controversial but is generally accepted as salvage therapy [5–7].

To get superior therapeutic results, combination modalities, such as the addition of ITS or HBOT to SS, have been tried by many authors, but various and conflicting results have been obtained [2,8–11]. Thus, Gau and Liu performed a metaanalysis with 8 studies about the addition of ITS to SS [12]. They reported better outcomes in pure tone threshold average (PTTA) scores, particularly in severe-profound hearing loss patients, with the addition of ITS. There are also 2 systematic reviews in the literature that were performed with 19 and 16 trials of the addition of HBOT to SS. Both of them recommended the addition of HBOT to SS, especially in severe-profound hearing loss [13,14]. 

In the literature, most of the studies compared the success rates of SS monotherapy with either ITS or HBOT. Only 2 studies compared the addition of ITS and HBOT to SS. Sukuzi et al. [15] concluded that the combination of ITS and SS was superior to the addition of HBOT in terms of recovery rate. However, Toroslu et al. [16] found no significant differences between the 2 combination protocols. Thus, we conducted this study firstly, to show the therapeutic superiority of the ITS or HBOT combination as initial therapy, and secondly, to exhibit the short-term and the long-term prognosis after the salvage treatment of the ISSHL patients.

## 2. Materials and methods

This clinical study with ISSHL patients was conducted in a tertiary referral center after the approval of local ethics committee (No: 42/13:06.11.2017) The recordings of these patients who were managed with different treatment protocols between 2012 and 2016 were evaluated retrospectively. 

### 2.1. Inclusion criteria

1. Patients who were hospitalized due to ISSHL with a symptom duration of fewer than 30 days.

2. Patients who completed the treatment protocols properly.

3. Patients who have the recordings of the pretreatment and posttreatment audiogram, otolaryngologic examination, and magnetic resonance imaging with contrast. 

### 2.2. Exclusion criteria 

1. Symptom duration of longer than 30 days. 

2. Previous history of Meniere’s disease, acoustic trauma, chronic otitis media, cerebellopontine angle pathologies, ototoxic drug usage, ISSHL, or otologic surgery.

3. Bilateral ISSHL.

4. Newly diagnosed vestibular schwannoma. 

5. Patients who were referred to our clinic after starting medication.

Patient characteristics including age, sex, affected ear side, presence of vertigo and tinnitus symptoms, history of systemic illness such as diabetes mellitus and hypertension, upper respiratory tract disease history, and time duration between the onset of symptoms and therapy were evaluated. 

According to the inclusion and exclusion criteria, the patients were divided into 3 groups for initial treatment. Group 1 was the steroid monotherapy group, and the treatment consisted of SS plus 5 mg/kg of intravenous dextran for 5 days, plus 3 × 1600 mg oral piracetam till the end of therapy. Methylprednisolone was used as a single bolus dosage of 250 mg for the first day, 150 mg for the second day, and 100 mg for the third day. After the third day, 1 mg/kg/d oral methylprednisolone was started and tapered 16 mg every 3 days. Those in Group 2o received the Group 1 treatment protocol, plus simultaneously 5 doses of 2 mg of intratympanic dexamethasone (0.5 mL from 4 mg/ml ampul form) once every 2 days. Those in Group 3 received the Group 1 treatment protocol plus 120 min of 2.5-atmosphere HBOT for 20 consecutive days. 

Pretreatment and posttreatment audiologic tests were analyzed. Speech discrimination scores (SDS) were noted, and PTTA was calculated as the average of thresholds at 0.5, 1, 2, and 4 kHz. Posttreatment improvement of PTTA results were analyzed according to Furuhashi criteria [17] (Table 1). 

**Table 1:** Furuhashi criteria for the assessment of audiologic hearing outcome.

	Criteria
Complete recovery	PTTA <20 dB or identical to contralateral nonaffected ear
Marked improvement	PTTA improvement >30 dB
Slight improvement	PTTA improvement between 10 and 30 dB
No recovery	PTTA improvement <10 dB

PTTA: Pure tone threshold average (500, 1000, 2000, and 4000 Hz)

The clinical records of the patients without complete recovery who were directed to salvage treatment were also analyzed. Group 1 patients were treated with ITS protocol and then HBOT if there was no complete recovery. In Group 2 and Group 3 patients, HBOT and ITS protocols were added as salvage, respectively. The pre- and postsalvage audiograms were compared. Finally, the last audiologic data of the patients after the follow-up were examined and compared with the postsalvage results.

### 2.3. Statistical analyses

Statistical analyses were performed with IBM SPSS 22.0 for Windows (IBM Corp., Armonk, NY, USA). Numerical variables were summarized as mean ± standard deviation or median (minimum–maximum). Categorical variables were given as frequencies and percentages. Categorical variables were compared by chi-square test. Normality of the continuous variables was evaluated by the Kolmogorov-Smirnov test. Homogeneity of variances was tested by Levene test. Differences between the groups according to continuous variables were determined by one-way ANOVA or Kruskal–Wallis test as appropriate. A P-value of less than 0.05 was considered significant.

## 3. Results

The study group was composed of 96 patients. All 3 groups had 32 patients each. Patient characteristics including age, sex, affected ear side, presence of vertigo and tinnitus symptoms, history of systemic illness such as diabetes mellitus and hypertension, upper respiratory tract disease history, and time duration between onset of symptoms and therapy are shown in Table 2. According to these characteristics, there was no statistically significant difference, so there was no heterogeneity in each group. 

**Table 2:** Patient characteristics of Groups 1, 2, and 3.

	Group 1(n: 32)	Group 2(n: 32)	Group 3(n: 32)	P- value
Age (mean ± standard deviation)	45.81 ± 19.45	48.62 ± 15.39	43.31 ± 12.32	0.322
Sex (male/female)	25/7	20/12	18/14	0.165
Side (right/left)	12/20	18/14	12/20	0.218
Presence of vertigo (yes/no)	6/26	3/29	5/27	0.542
Presence of tinnitus (yes/no)	24/8	22/10	21/11	0.708
Presence of diabetes mellitus (yes/no)	5/27	8/24	4/28	0.395
Presence of hypertension (yes/no)	8/24	9/23	4/28	0.278
Presence of upper respiratory tract infection (yes/no)	2/30	2/30	3/29	0.862
Time interval between onset of symptom and therapy (days) (mean ± standard deviation)	4.5 ± 4.52	5.22 ± 3.3	4.09 ± 4.02	0.079

The groups were also investigated according to pretreatment audiologic data. The level of hearing loss of each patient was categorized according to initial PTTA at 500, 1000, 2000, and 4000 Hz, and classified as mild (26–40 dB), moderate (41–60 dB), severe (61–80 dB), and profound (>80 dB) hearing loss. Also, PTTA and SDS were calculated in each group. All groups were statistically homogenous concerning initial audiologic assessment, which is shown in Table 3. 

**Table 3:** Pretreatment audiologic assessment of Groups 1, 2, and 3.

	Group 1(n: 32)	Group 2(n: 32)	Group 3(n: 32)	P-value
Audiogram shape (number of patients)				
Ascending	11	9	12	0.805
Flat or deaf	18	17	16	
Descending	3	6	4	
Pretreatment degree of hearing loss (n)				
Mild	13	5	4	0.153
Moderate	7	10	12	
Severe	4	8	7	
Profound	8	9	9	
Pretreatment PTTA (dB) (mean ± standard deviation)	57.44 ± 30.37	66.09 ± 25.06	66.37 ± 24.63	0.321
Pretreatment SDS (%) (mean ± standard deviation)	60.5 ± 37.6	48.62 ± 32.16	48.87 ± 31.82	0.089

PTTA: Pure tone threshold average (500, 1000, 2000, and 4000 Hz) dB: Decibels, SDS: Speech discrimination score

The audiologic evaluation after initial treatment was evaluated, and PTTA of all patients in each groups was categorized according to Furuhashi criteria. The results are shown in Table 4. There was no statistically significant difference in posttreatment audiologic results between the groups (P: 0.66). Complete recovery and marked improvement were accepted as successful treatment, and was the result for 24 (75%), 18 (56,3%), and 19 (59.4%) patients in Groups 1, 2, and 3, respectively. There was no significant difference between groups (P: 0.248) (Table 5). The overall success rate was 63.5%, and the overall complete recovery rate after initial treatment was 50%, regardless of the type of treatment regimens in all 96 patients.

**Table 4:** Posttreatment categorization according to Furuhashi criteria.

Number of patients (%)	Group 1	Group 2	Group 3	P-value
Complete recovery	19 (59.4%)	14 (43.8%)	15 (46.9%)	0.66
Marked improvement	5 (15.6%)	4 (12.5%)	4 (12.5%)	
Slight improvement	3 (9.4%)	8 (25%)	5 (15.6%)	
No recovery	5 (15.6%)	6 (18.7%)	8 (25%)	
Total	32	32	32	

**Table 5:** Results of successful treatment after initial therapy in Groups 1, 2, and 3.

	Mean	Standard deviation	P-value
Presalvage PTTA	70.56	24.77	0.121
Postsalvage PTTA	65.14	24.60	
Long-term follow-up PTTA	68.52	24.43	0.223

* Successful criteria: Total number of patients in complete recovery and marked improvement groups according to Furuhashi criteria.

The patients without complete recovery (n: 48, 50%) were directed to salvage therapy. Only 40 of these patients’ audiologic results were available at the end of salvage treatment. Presalvage and postsalvage PTTA results were evaluated, and the patients were categorized according to Furuhashi criteria. Only 3 of the patients (2 patients with complete recovery, 1 patient with marked improvement) out of 40 (7.5%) obtained successful results after salvage treatment. Thus, the success rate was 58% after the completion of the initial treatment and salvage treatment in 88 patients. The audiograms of these 40 patients were reanalyzed with a mean of 36.5 (±18.61) months of follow-up after the end of salvage treatment. There was no statistically significant difference in PTTA between presalvage and postsalvage or between postsalvage and long-term follow-up (P: 0.121, P: 0.223, respectively) (Table 6). The long-term follow-up spontaneous recovery rate after the salvage therapy with a mean follow-up of 36.5 months was 0%. 

**Table 6:** Comparison of presalvage, postsalvage, and long-term follow-up PTTA.

	Group 1	Group 2	Group 3	P-value
Successful* (Patient no. %)	24 (75%)	18 (56.3%)	19 (59.4%)	0.248
Unsuccessful (Patient no. %)	8 (25%)	14 (43.7%)	13 (40.6%)	
Total	32	32	32	

PTTA: Pure tone threshold average (500, 1000, 2000, and 4000 Hz)

## 4. Discussion

Due to the lack of proven definitive etiopathogenetic mechanisms in ISSHL, no standard 100% curative treatment modality has been established yet. The recovery rate of patients with ISSHL, regardless of therapy regimens, was shown to be up to 89% [18]. There are several prognostic factors influencing the hearing outcome. In addition to the type of treatment protocol, success also depends on age, presence of tinnitus, initial hearing level, smoking, the shape of audiometry curve, and the time interval between the onset of symptoms and the beginning of therapy [19–21]. Furthermore, there is still a lack of consensus in evaluation criteria of hearing outcome. Pure tone audiometry threshold levels in dB values, SDS, or categorizations according to hearing improvement such as Furuhashi criteria are generally used for hearing outcome assessment [22]. Another point to mention is that there are still no uniform standards in dosage and duration of protocols, regardless of any sort of therapy. Because of these reasons, different and conflicting results are found in the literature. Although Cinamon et al. [23] found that there were no significant difference in recovery rate between groups receiving SS treatment and a placebo, Wilson et al. [24] reported significant improvement of SS over a placebo. In our study, the groups were homogenous according to prognostic factors, and the complete recovery rate was the highest in the SS monotherapy group (59.4% vs. 43.8% and 46.9%). In Group 1, better but insignificant pretreatment PTTA and SDS levels may have led to higher success rates compared to the other groups. 

Systemic usage of steroids has some limitations due to serious side effects such as peptic ulcer, glaucoma, uncontrolled diabetes mellitus, hypertension, osteoporosis, and avascular necrosis of the femur. So, the intratympanic route has become popular both in salvage and initial treatment of ISSHL. Successful results were shown by several studies in both initial treatment and salvage therapy after SS [18,24–27]. In the present study, ITS in combination with SS as an initial treatment resulted in a complete recovery rate of 43.8%. In the literature, initial ITS treatment in ISSHL has a success rate range between 55% and 80% [28]. The recommended time duration between symptom onset and ITS therapy to best improve the outcome is fewer than 10 days [22]. Although our mean duration of initiation of ITS therapy was 5.2 days, we weren’t able to achieve better results with the addition of ITS. The addition of ITS to SS has revealed different results in the literature. Battaglia et al. [25] and Gundogan et al. [29] reported better hearing improvement in ITS in combination with SS according to PTTA. On the other hand, Baysal et al. [30] and Koltsidopoulos et al. [31] concluded no superior hearing improvement of ITS in combination with SS. 

The usage of HBOT as an initial treatment of ISSHL was investigated by several authors. Cekin et al. [32] and Alimoglu et al. [33] compared HBOT plus oral steroids with treatment by oral steroids alone retrospectively and found no significant differences between them. Topuz et al. [5] and Fujimura et al. [34] reported significant improvement in hearing outcome with HBOT as the initial treatment of ISSHL. Thus there is still controversy about the effectiveness of HBOT in initial management. Our study showed that the addition of HBOT to SS as initial treatment did not result in better hearing outcome compared to SS monotherapy. 

There are only 2 studies comparing ITS in combination with HBOT to SS as an initial treatment in the literature. Suzuki et al. [15] compared 174 patients who received ITS plus SS with 102 patients who received HBOT plus SS, retrospectively. The total recovery (cure) rate was 29.3% and 21.6% with the combination of ITS and HBOT, respectively, which was not statistically significantly different. Although the group sizes were much larger than in our study, the groups were not homogenous according to initial hearing level and presence of vertigo, and there was no control group receiving SS monotherapy. They concluded that the addition of ITS probably leads to a higher recovery rate than the addition of HBOT. Toroslu et al. [16] analyzed the combination of ITS and HBOT with SS with 90 patients retrospectively. The study consisted of 4 groups: Group 1 received oral steroids, Group 2 received oral steroids and ITS, Group 3 received oral steroids and HBOT, and Group 4 received ITS. The overall complete recovery rate of all 90 patients was 32.2%, which was lower than in our study. They compared the mean hearing gain in dB instead of the recovery rate. There was no statistically significant difference in hearing gain within subgroups. Our result was similar to the aforementioned 2 studies in that neither ITS nor HBOT in addition to SS had a superior recovery rate in ISSHL as initial treatment. 

It is important to establish the clinical progress and long-term prognosis of ISSHL. Intratympanic steroids and HBOT are commonly used as salvage treatment after SS therapy failure and various results have been reported [20,35]. Most of the patients recover within the first month of therapy [36,37]. In a retrospective study, delayed recovery (21.8%) occurred after the first month of discharge, and the complete recovery rate was 4.6% [37]. In our study, the complete recovery rate after salvage therapy was 5% (2 patients). As hearing recovery generally occurs within the first month of therapy, treatment should be done within the first 30 days to gain maximal hearing outcome. Although the salvage group size (40 patients) was not large enough, the present study showed that there were no significant spontaneous recovery or deterioration in PTTA after the salvage treatment with a mean follow-up of 36.5 months. 

This study has some limitations. Due to the low incidence of ISSHL, the number of patients in this retrospective study was not large enough, and there was no placebo subgroup. Secondly, a smoking habit, which is a poor prognostic factor, was not evaluated in patient characteristics. Thirdly, there was no hearing outcome analysis with respect to pretreatment hearing loss degree. Lastly, posttreatment levels of SDS and frequency-specific pure tone thresholds and mean hearing gain in dB were not evaluated in the assessment of outcome. 

 In conclusion, the addition of ITS or HBOT to SS for ISSHL as initial treatment did not cause statistically significant therapeutic superiority and improvement. Furthermore, the efficacy in hearing improvement of salvage treatment in ISSHL is still limited. No spontaneous recovery occurs after the end of salvage treatment during long-term follow-up. Thus, for ISSHL and many inner ear diseases, the cochlea is still a mysterious locked box of which definitive pathogenesis has not been clearly explored yet. Between several types of treatment modalities, only steroids and hyperbaric oxygen were shown to be effective in ISSHL. Yet there is no alternative treatment protocol superior to these. New regimens should be introduced after clarification of definitive etiopathogenesis by the help of studies in the future. 

## Acknowledgement/Disclaimers/Conflict of interest

All authors confirm that there are no conflicts of interest associated with this publication and there has been no significant financial support for the study.

## Informed consent

This study was conducted after all patients signed informed consent and after the approval of institutional review board (No:42/13:06.11.2017).
